# Supplementary material for: From Iron to Copper: The Effect of Transition Metal Catalysts on the Hydrogen Storage Properties of Nanoconfined LiBH4 in a Graphene-Rich N-Doped Matrix
Source: Molecules. 2022 May 3;27(9):2921. doi: 10.3390/molecules27092921 (PMC9103407; doi:10.3390/molecules27092921)
Supplement: Supplementary file 1 [file molecules-27-02921-s001.zip › Molecule 2022 SI 3.pdf]

# From iron to copper: the effect of transition metal catalysts on the hydrogen storage properties of nanoconfined $LiBH_4$ in a graphene-rich N-doped matrix.

**Alejandra A. Martínez [1,2], Aurelien Gasnier [1,2,*] and Fabiana C. Gennari [1,3]**

[1] Consejo Nacional de Investigaciones Científicas y Técnicas (CONICET) and Centro Atómico Bariloche (CNEA), Av. Bustillo 9500, R8402AGP, S. C. de Bariloche, Río Negro, Argentina; andreaalejandra.m5@gmail.com (A.M.); gennari@cab.cnea.gov.ar (F.G.)

[2] Instituto de Nanociencia y Nanotecnología, S. C. de Bariloche, Río Negro, Argentina

[3] Instituto Balseiro, Universidad Nacional de Cuyo, Argentina

* Correspondence: aurelien.gasnier@cab.cnea.gov.ar; Tel.: +54-294-444-5556

Supplementary Information

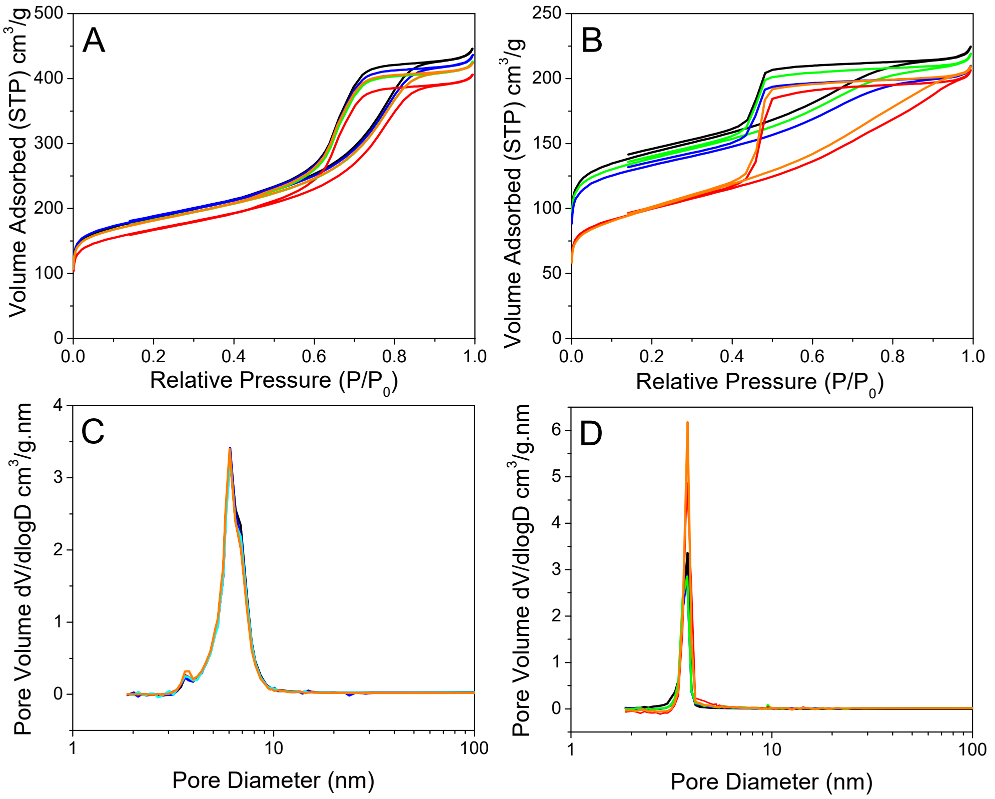


**Figure SI 1.** Nitrogen isotherms (A, B) and corresponding pore size distribution obtained by BJH (C, D) of resins GN (A, C, black) and G2N (B, D, black) decorated with Fe (orange), Co (red), Ni (green) and Cu (blue).

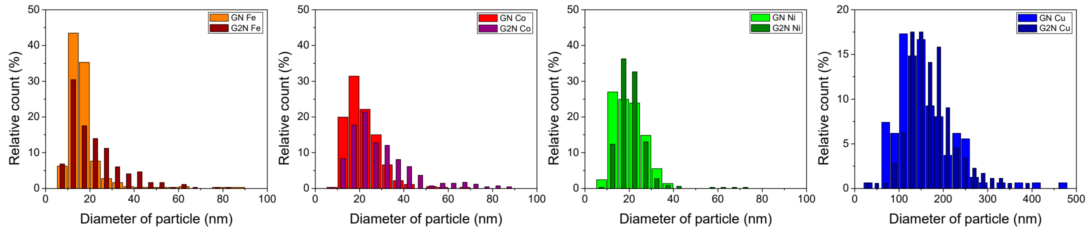


**Figure SI 2.** Size distribution histograms of metallic nanoparticles observed by SEM of non-impregnated matrices GN (light) and G2N (dark) decorated with Fe (orange, brown), Co (red, wine), Ni (green, dark green), Cu (blue, deep blue).

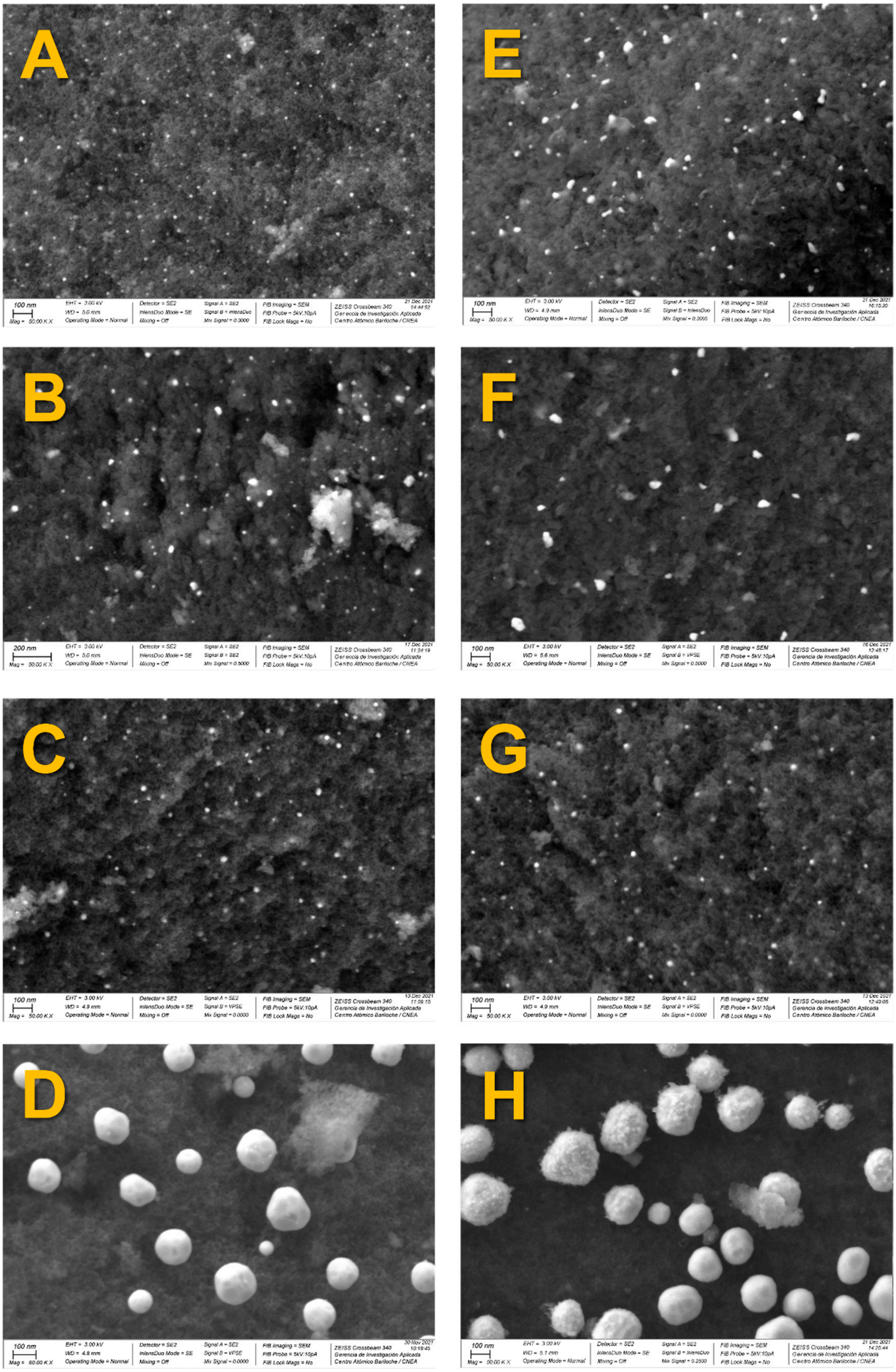


**Figure SI 3.** High magnification (50k x) SEM observations of GN (A, B, C, D) and G2N (E, F, G, H) free matrixes decorated with Fe (A, E), Co (B, F), Ni (C, G), and Cu (D, H).

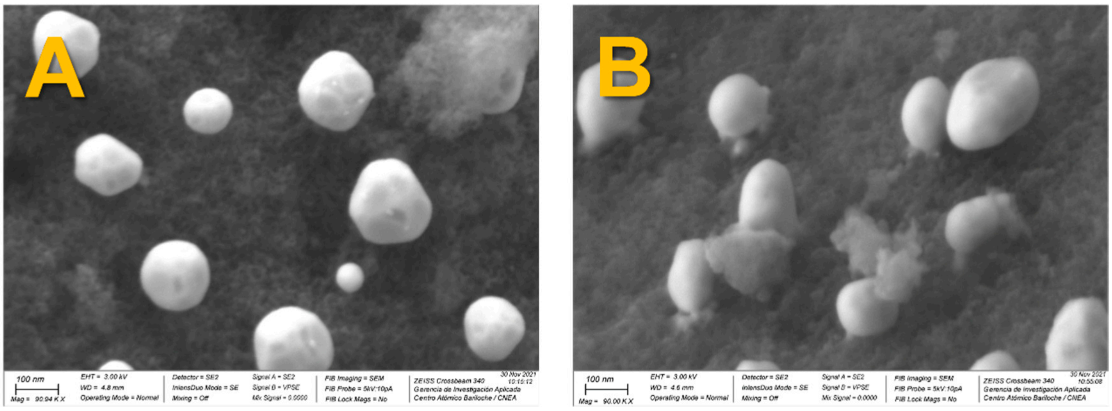


**Figure SI 4.** Very high magnification (90 k x) SEM observations of Cu nanoparticles decorating GN (A) and G2N (B) matrixes.

The impact of the matrix over the particle´s shape is displayed by their rounder shape over G2N, particularly their “melted” appearance at the matrix contact.

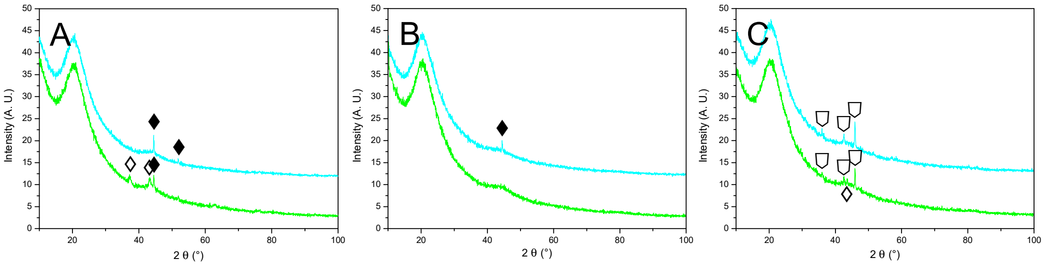


**Figure SI 5.** PXRD traces of free (A), just impregnated (B) and cycled (C) GN matrixes decorated with Ni nanoparticles prepared by incipient wetness impregnation in methanol (green) versus manual grinding (teal) of nitrates. The symbols are attributed to the cubic metal (♦), its oxide (◊), and the metal boride (🛡).

It confirms that nickel oxide nanoparticles observed in GN Ni originated from the incipient wetness impregnation method. After impregnation (B), the peak attributed to metallic nickel disappeared in the material prepared by incipient wetness but remained visible in the material prepared by manual grinding. After cycling (C) the materials obtained by incipient wetness impregnation present peaks attributed to $Ni_2B$ but also very limited quantities of NiO.

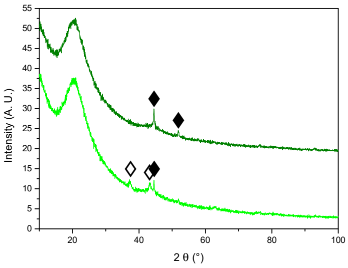


**Figure SI 6.** PXRD traces of GN Ni just prepared by incipient wetness impregnation in methanol (green) and after 30 min at 300 ºC, 60 atm $H_2$ (dark green).

It illustrates that the oxides (◊) originally observed after pyrolysis are reduced to cubic metals (♦) under the conditions of impregnation, even in the absence of $LiBH_4$.

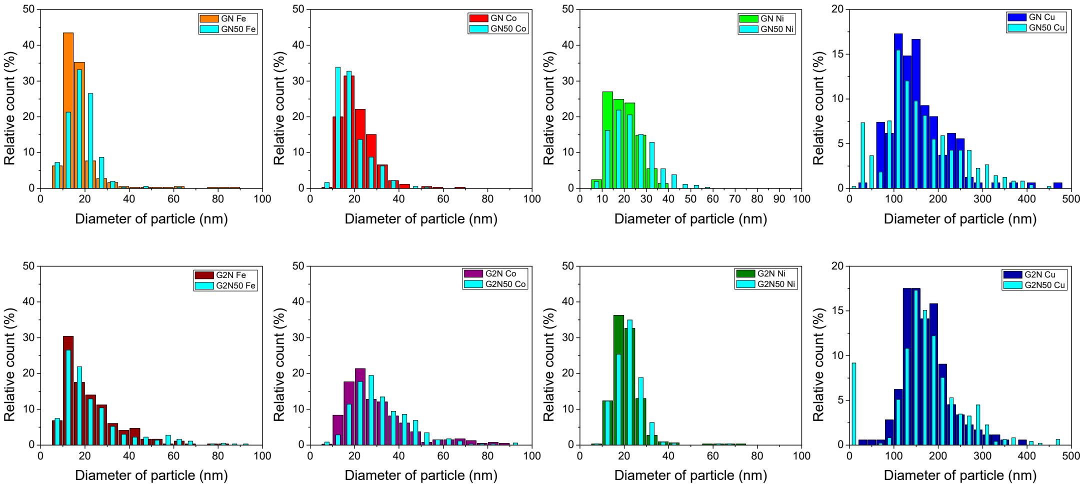


**Figure SI 7.** Size distribution histograms of metallic nanoparticles observed by SEM of just impregnated matrices (cyan) GN50 and G2N50 with respect to their non-impregnated counterparts decorated with Fe (orange, brown), Co (red, wine), Ni (green, dark green), Cu (blue, deep blue).

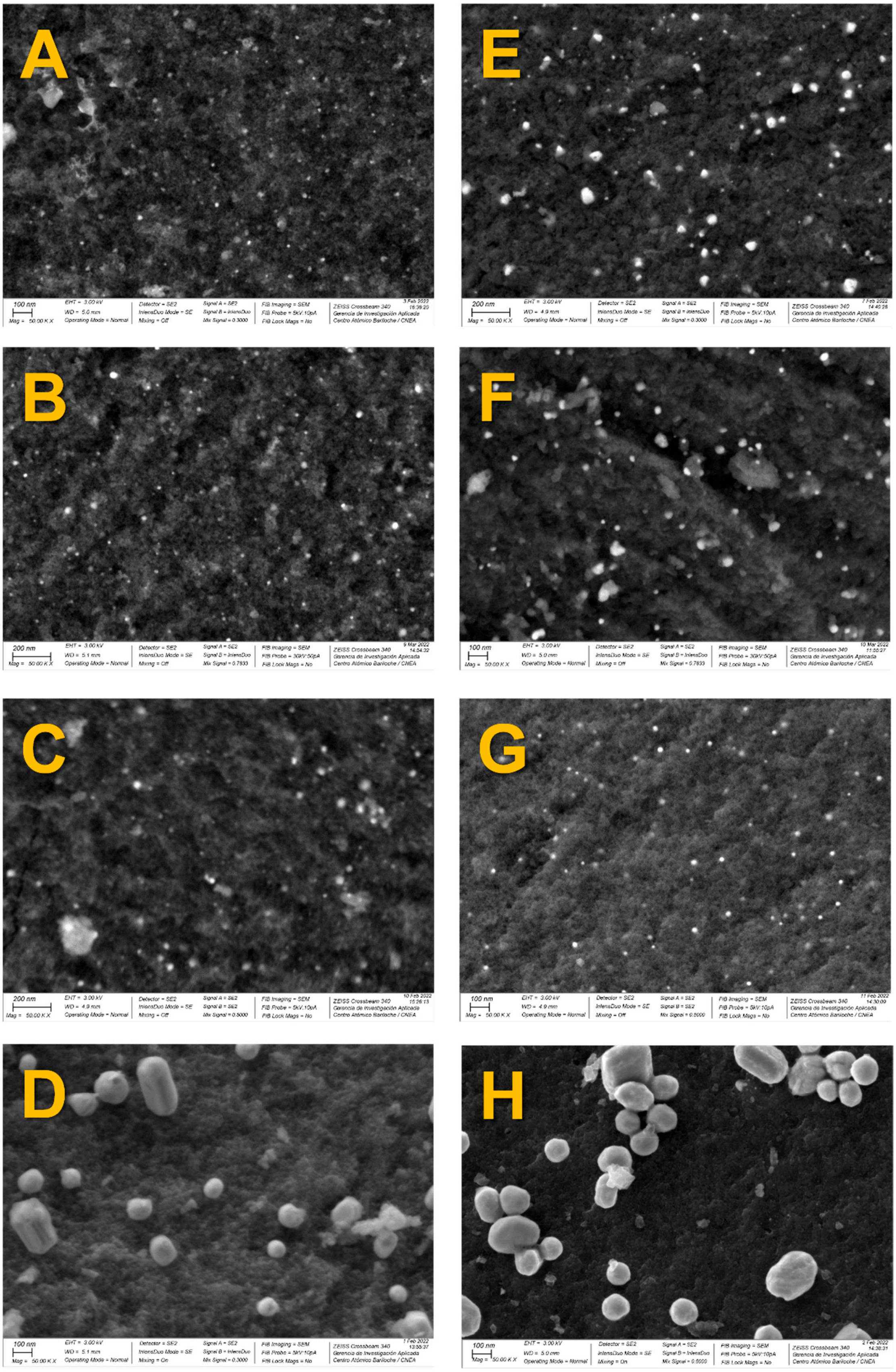


**Figure SI 8.** High magnification (50k x) SEM observations of GN50 (A, B, C, D) and G2N50 (E, F, G, H) just-impregnated matrixes decorated with Fe (A, E), Co (B, F), Ni (C, G), and Cu (D, H).

The nanoparticles present a more diffuse shape, probably due to LiBH4 derivative joining the matrix with the nanoparticle, sometime "gluing" several nanoparticles together. This is especially visible for G2N50 Cu (H).

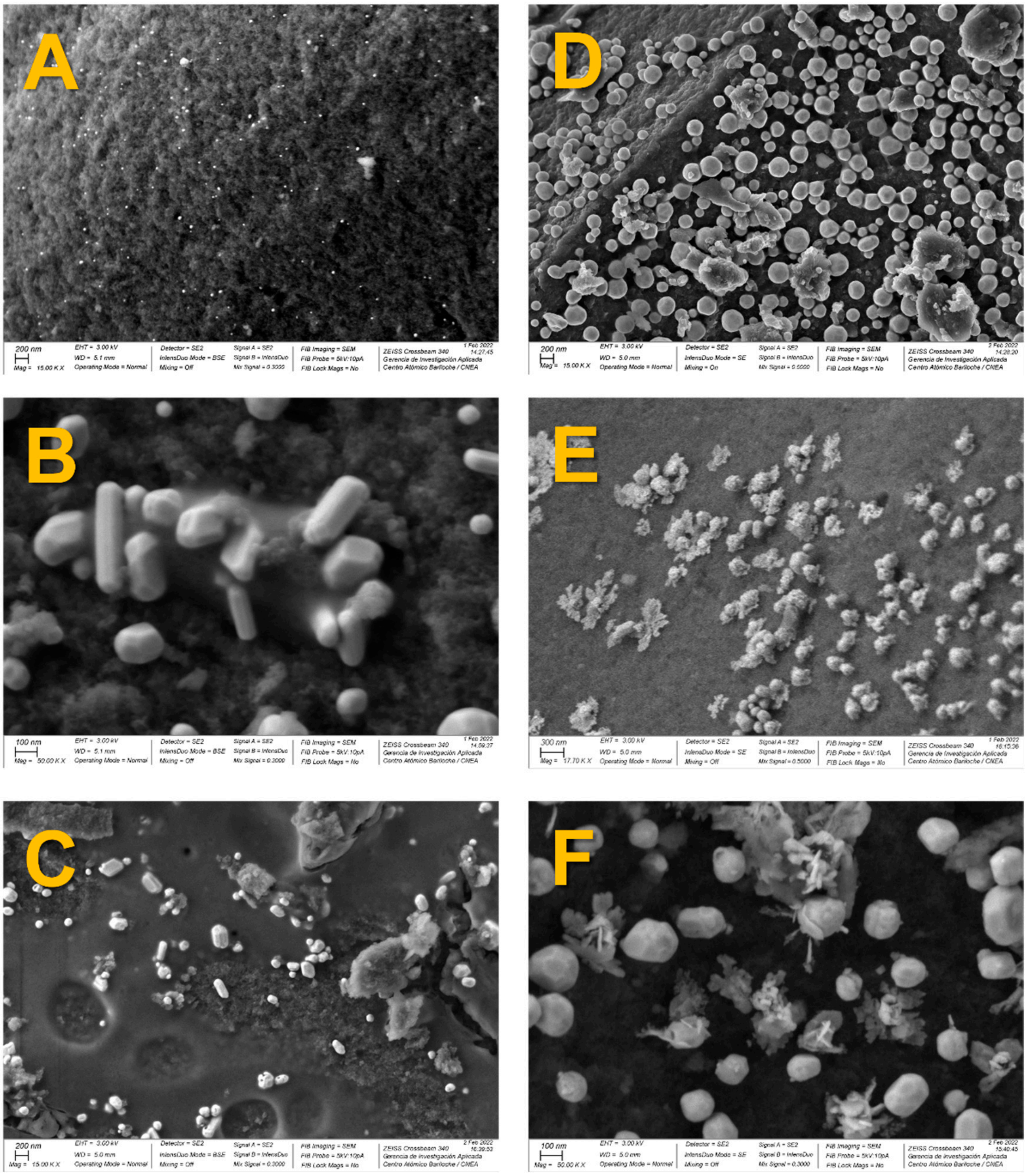


**Figure SI 9.** Cu decorated GN50 (A, B, C) and G2N50 (D, E, F) just-impregnated matrixes.

Some regions presented a remarkable coverage of small-sized Cu-nanoparticles (A). When excess $LiBH_4$ was observed, the nanoparticles displayed a tendency to bond together in a group, glued with $LiBH_4$ derivative (B, C, D). G2N50 Cu display some splashed Cu material (E, F)

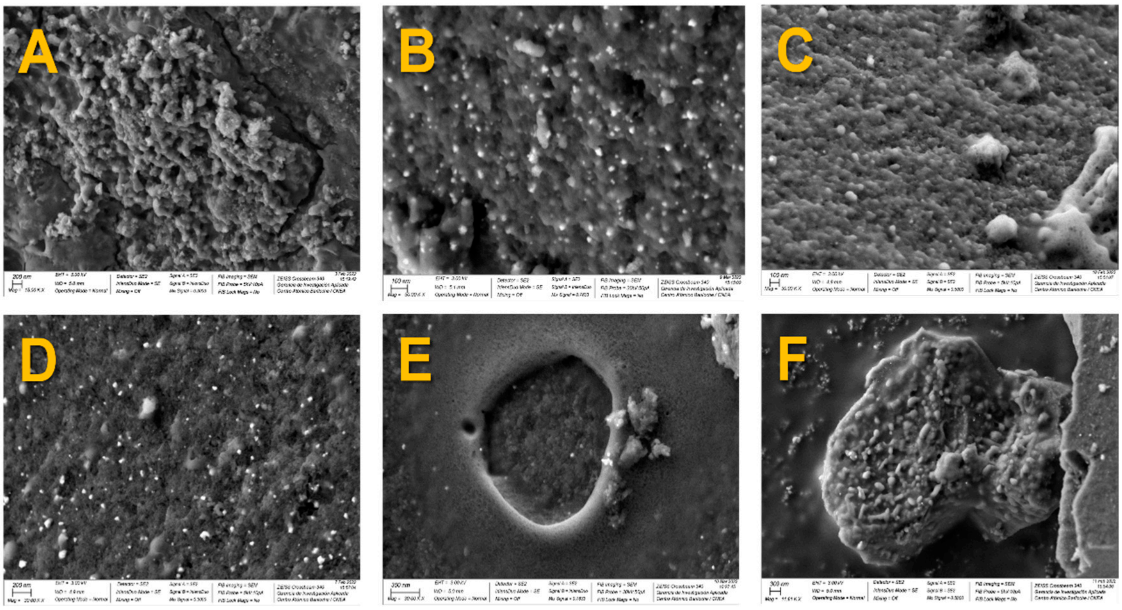


**Figure SI 10.** GN50 (A, B, C) and G2N50 (D, E, F) just-impregnated matrixes decorated with Fe (A, D), Co (B, E) and Ni (C, F) displaying excessive $LiBH_4$ derivative with nanoparticles visible by transparency.

A) $LiBH_4$ derivative developed around the matrix frame, and some nanoparticles can be seen in transparency.
B) $LiBH_4$ derivative is present in even more excess, the surface being here barely molten.
C) Even more $LiBH_4$ is covering the surface of the matrix, flattening it.
D) An alternance of filled and unfilled matrix can be observed here with some blurry spots.
E) Very excessive amount of $LiBH_4$ derivative, resulting in a smooth surface.
F) Excessive amount of $LiBH_4$ derivative, with many molten structures.

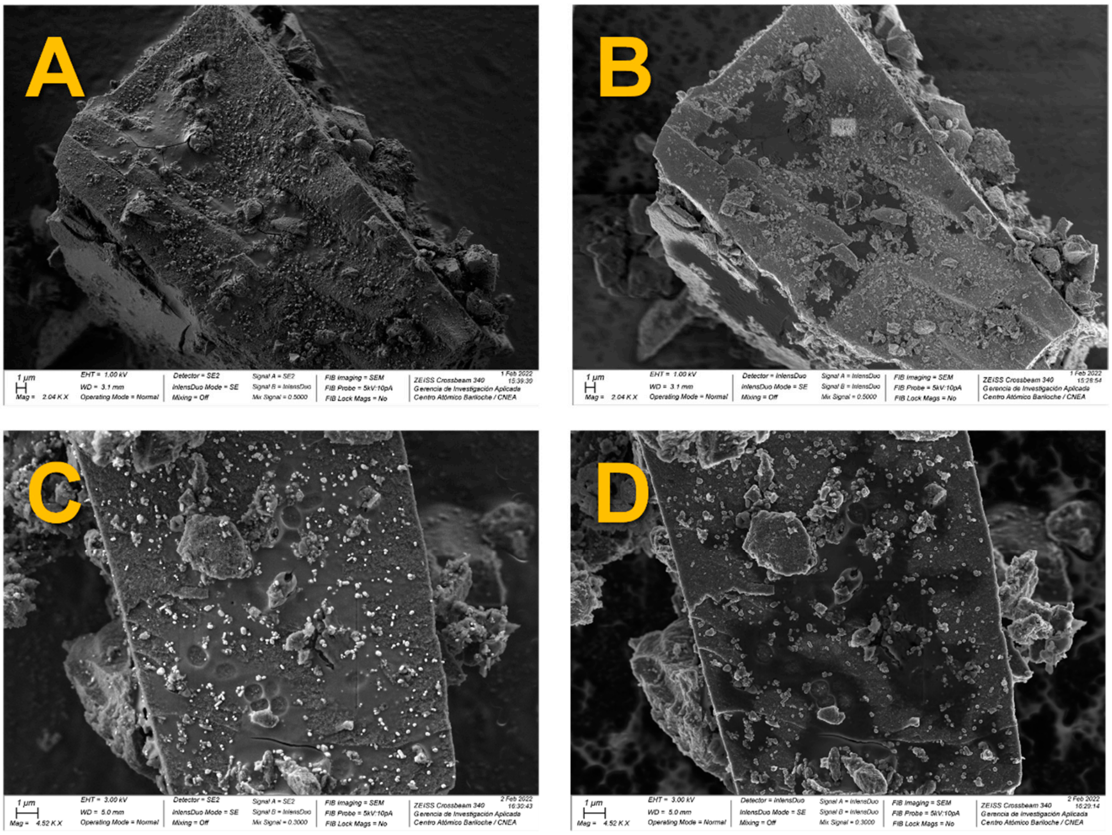


**Figure SI 11.** Just-impregnated decorated matrix with excessive $LiBH_4$ derivative observed by secondary electrons (A, C) and in-lens (B, D) modes.

Some particles displayed an unusual proportion of $LiBH_4$ when the matrix was covered with metallic nanoparticles. While the contrast between the matrix and the $LiBH_4$ derivative is not strong with secondary electrons (A, C), the in-lens mode (B, D) was much more efficient at resolving those elements, and the $LiBH_4$ derivatives appeared darker than their surroundings. We are not sure what is responsible for this behaviour but we suggest that the boron degradation product might absorb more electrons than carbon (which explain it appears darker when observed vertically), while electrons can bounce without difficulties in both (which explain no difference is observed at secondary electrons angle).

Cracks are particularly present when excess boron material was observed, suggesting that the hydrolysis of $LiBH_4$ promotes a fast expanse of the material, resulting in its ejection from the matrix and breaking it.

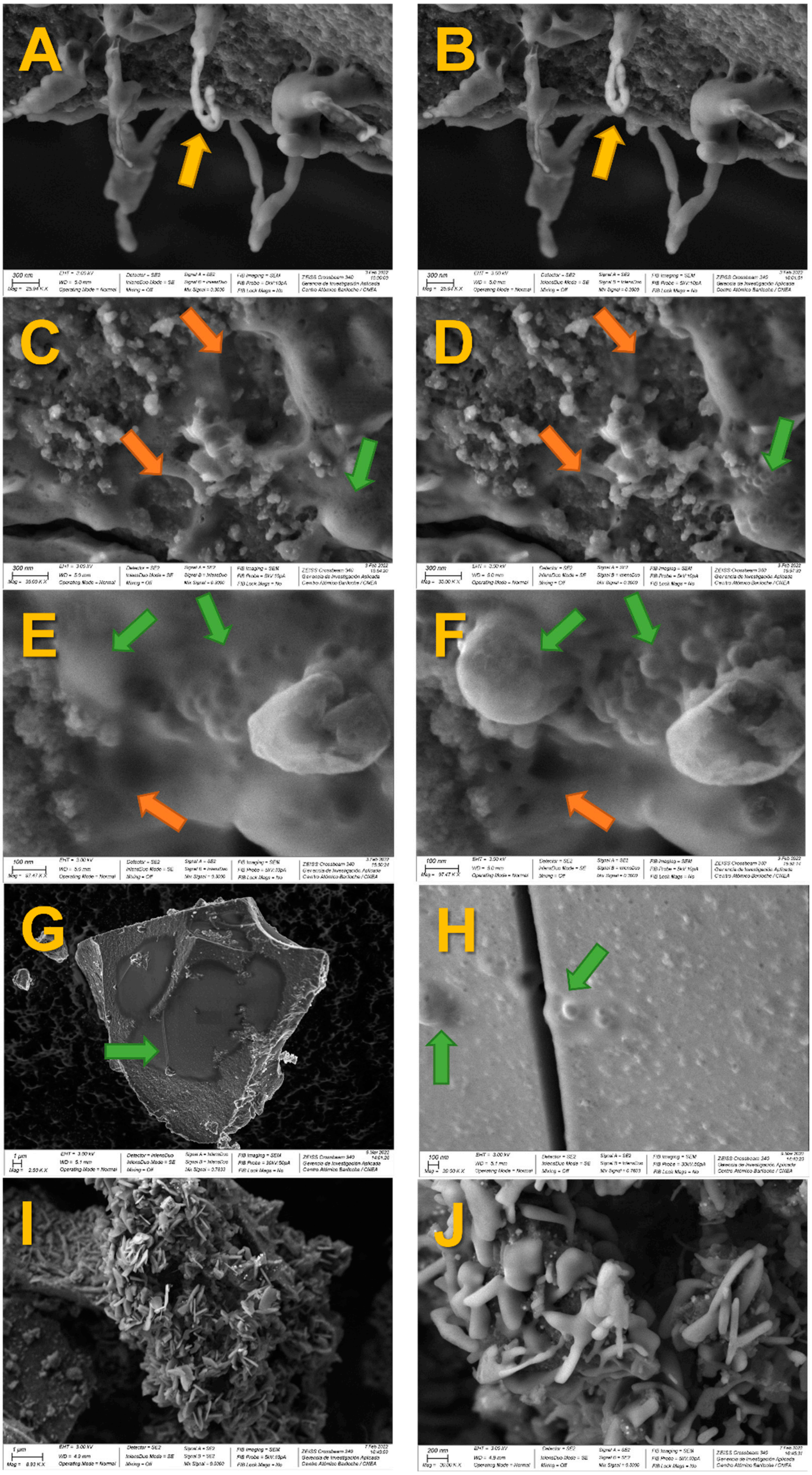


**Figure SI 12.** Evolution of excess $LiBH_4$ degradation products in just impregnated matrixes (A, C, E, G, I), and after several minutes of observation (B, D, F, H, J).

$LiBH_4$ derivatives were observed forming a strand-type structure (A, yellow arrow) that curled after some scan (B). Large excess $LiBH_4$ was observed (B, E, G) presenting some rugosity, probably of metallic nanoparticles, and forming cavities (orange arrows) or flat surfaces (green arrows). After scanning (D, F, H) the cavities the underlying matrix started to appear while plain surfaces were covered with excrescence. In rare occasion (I) a particle was covered with sharp flat structures of $LiBH_4$ degradation product, that started to melt upon scanning (J). The boron-based materials were sensible to the observation, their structure being affected by short observation time. Flat structures were covered with excrescences, indentations revealed the underlying matrix, and in some case a curling strand was observed. This drove use to suggest that electrons are absorbed by the boron material, which increase locally its temperature, promoting its fusion and triggering its reinsertion within the matrix.

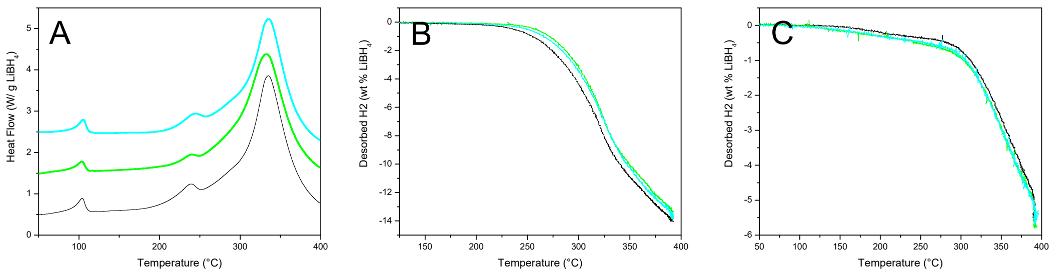


**Figure SI 13.** Calorimetric (A) and volumetric (B: first cycle, C: second cycle) studies of GN50 without (black) and with Ni nanoparticles formed by incipient wetness impregnation (green) and manual grinding (teal).

It appears that the decoration method has very little effect on the functional properties of the material.

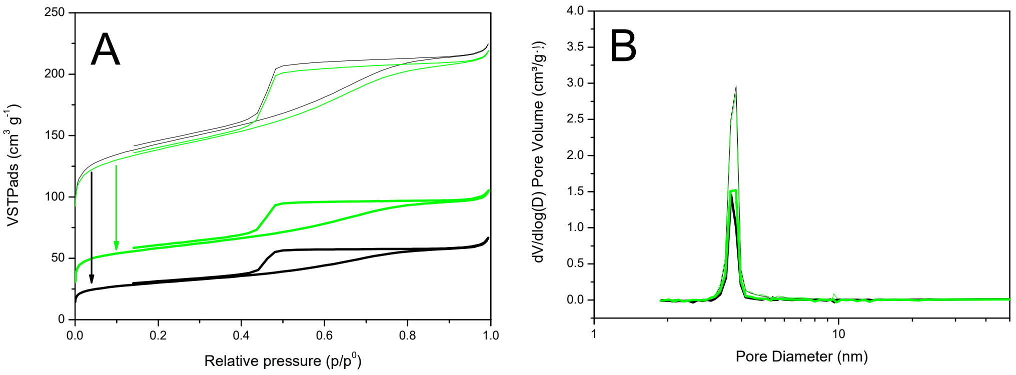


**Figure SI 14.** A) Isotherms of G2N (black) and G2N Ni (green) before (up, thin) and after (down, bold) impregnation at 50 vol. % with $LiBH_4$. B) pore-size distribution obtained by BJH of G2N (black) and G2N Ni (green) before (up, thin) and after (down, bold) impregnation at 50 vol. % with $LiBH_4$.

It can be observed that while the presence of Ni nanoparticles has no effect over the filling of the mesopores, the presence of Ni nanoparticles hinders the filling of the micropores.

| Matrix type | Metallic NP | $S_{BET}$ [a] ($m^2/g$) | $S_{ext}$ [b] ($m^2/g$) | $S_{micro}$ [c] ($m^2/g$) | $V_{tot}$ [d] ($cm^3/g$) | $V_{meso}$ [e] ($cm^3/g$) | $V_{micro}$ [c] ($cm^3/g$) | $D_{max}$ [e] (nm) |
|---|---|---|---|---|---|---|---|---|
| G2N | None | 580 | 190 | 390 | 0.35 | 0.17 | 0.16 | 3.8 |
| | Ni | 520 | 180 | 340 | 0.32 | 0.17 | 0.14 | 3.8 |
| G2N50 | None | 110 | 60 | 50 | 0.09 | 0.07 | 0.02 | 3.8 |
| | Ni | 210 | 90 | 120 | 0.15 | 0.09 | 0.06 | 3.8 |

Values determined by [a] BET, [b] BET-t-plot, [c] t-plot, [d] Gurvich, [e] BJH.

**Table SI 1.** Textural parameters of G2N before and after impregnation of $LiBH_4$ at 50 vol. % with and without Ni nanoparticles.

While 50 vol % of $LiBH_4$ filled 90 % of G2N´s micropores, in the presence of Ni nanoparticles only 60 % of the micropores were filled. 60 % of the mesopores were filled when Ni was absent while it reduced to 50 % when Ni was present.  It indicates that Ni hinders the wetting of the smaller pores by $LiBH_4$.
