# Supplementary material for: From Iron to Copper: The Effect of Transition Metal Catalysts on the Hydrogen Storage Properties of Nanoconfined LiBH4 in a Graphene-Rich N-Doped Matrix
Source: Molecules. 2022 May 3;27(9):2921. doi: 10.3390/molecules27092921 (PMC9103407; doi:10.3390/molecules27092921)
Supplement: Supplementary file 1 [file molecules-27-02921-s001.zip › Molecule 2022 SI Co Map.pdf]

From iron to copper: the effect of transition metal catalysts on the hydrogen storage properties of nanoconfined $LiBH_4$ in a graphene-rich N-doped matrix.

Alejandra A. Martínez [1,2], Aurelien Gasnier [1,2,*] and Fabiana C. Gennari [1,3]

1 Consejo Nacional de Investigaciones Científicas y Técnicas (CONICET) and Centro Atómico Bariloche (CNEA), Av. Bustillo 9500, R8402AGP, S. C. de Bariloche, Río Negro, Argentina; andreaalejandra.m5@gmail.com (A.M.); gennari@cab.cnea.gov.ar (F.G.)
2 Instituto de Nanociencia y Nanotecnología, S. C. de Bariloche, Río Negro, Argentina
3 Instituto Balseiro, Universidad Nacional de Cuyo, Argentina
* Correspondence: aurelien.gasnier@cab.cnea.gov.ar; Tel.: +54-294-444-5556

elemental mapping of Co-decorated matrixes (SI Co Map)

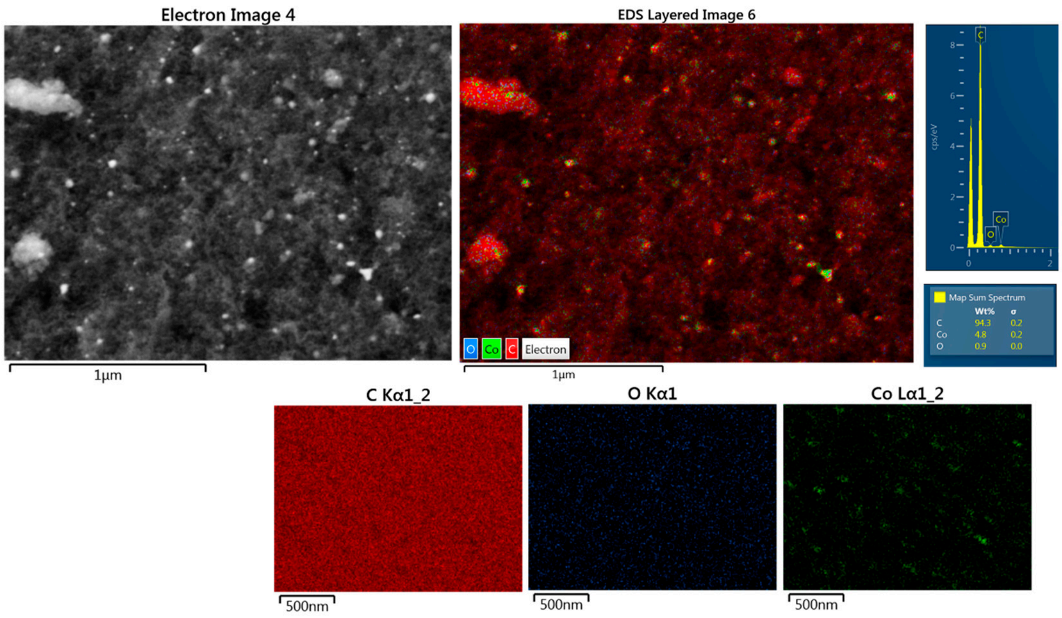

Figure SI Co 1. Elemental mapping of GN Co. A mixture of very small and medium nanoparticles can be observed, with an elemental distribution of Co very close to 5 wt. %.

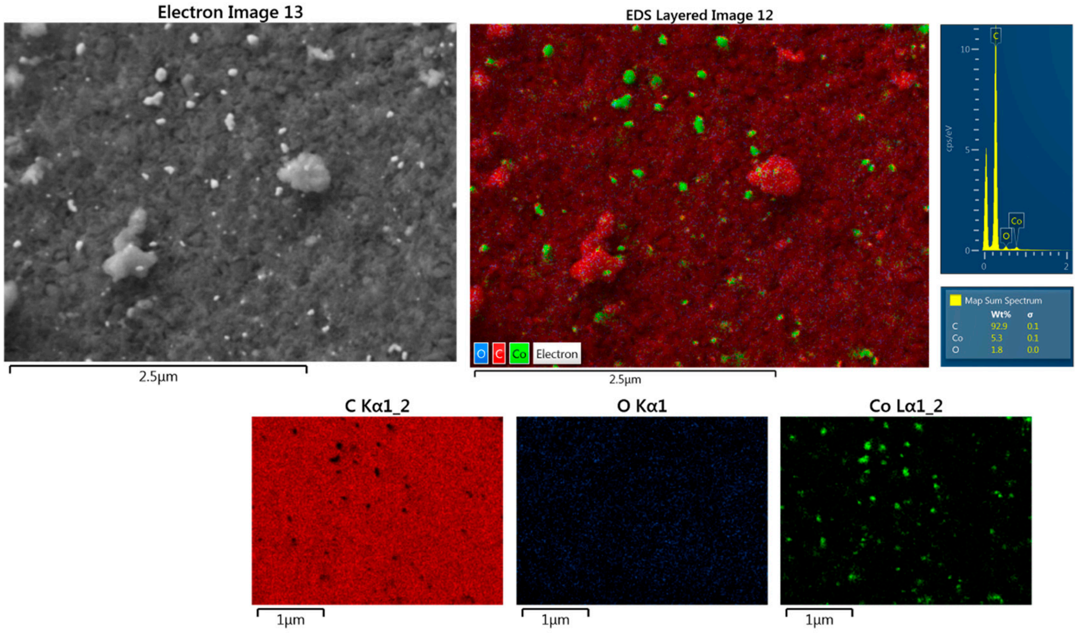

Figure SI Co 2. Elemental mapping of G2N Co. The distribution of particles is broader than in GN Co, with still small particles, but also bigger particles. Some Co particles display very blurry edges. The elemental distribution of Co is very close to 5 wt. %.

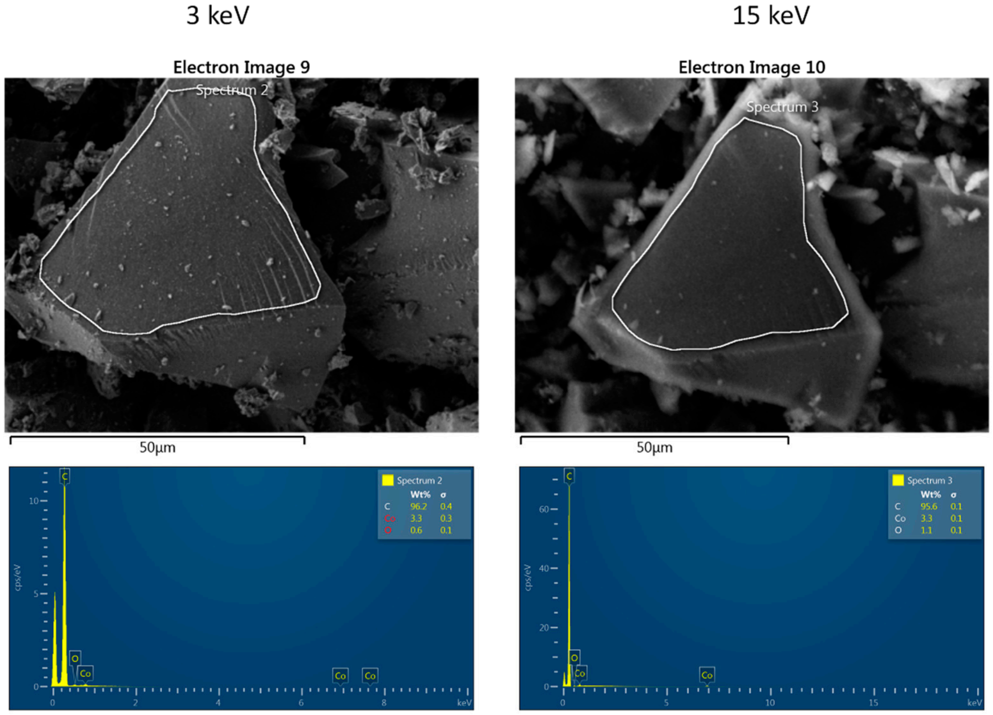

Figure SI Co 3. Elemental distribution of Co over a large area at 3 keV (left) and 15 keV (right). While the proportion of elemental Co is a bit lower than expected (3.3 wt. %), it isn´t affected by the beam´s energy, which is a good indication that the Co nanoparticles are evenly distributed along the depth of the matrix.

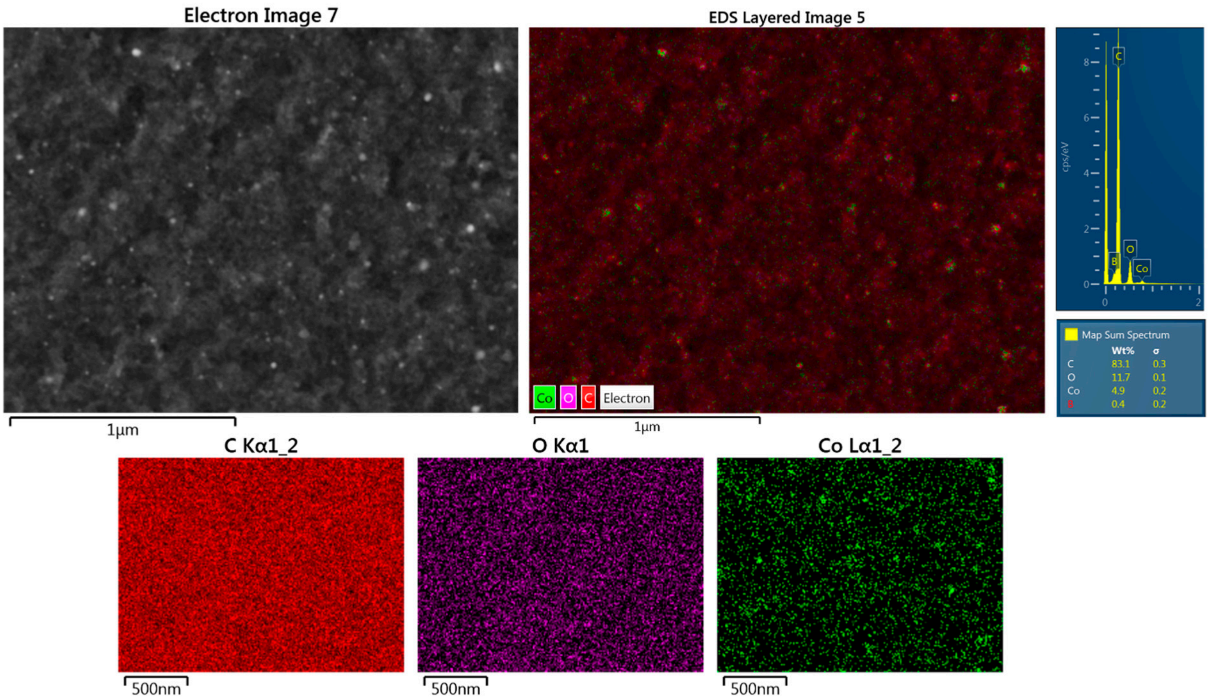

Figure SI Co 4. Elemental mapping of GN50 Co. A lot of small particles can be observed, with some particles of much bigger size. The elemental distribution is very close to 5 wt. %.

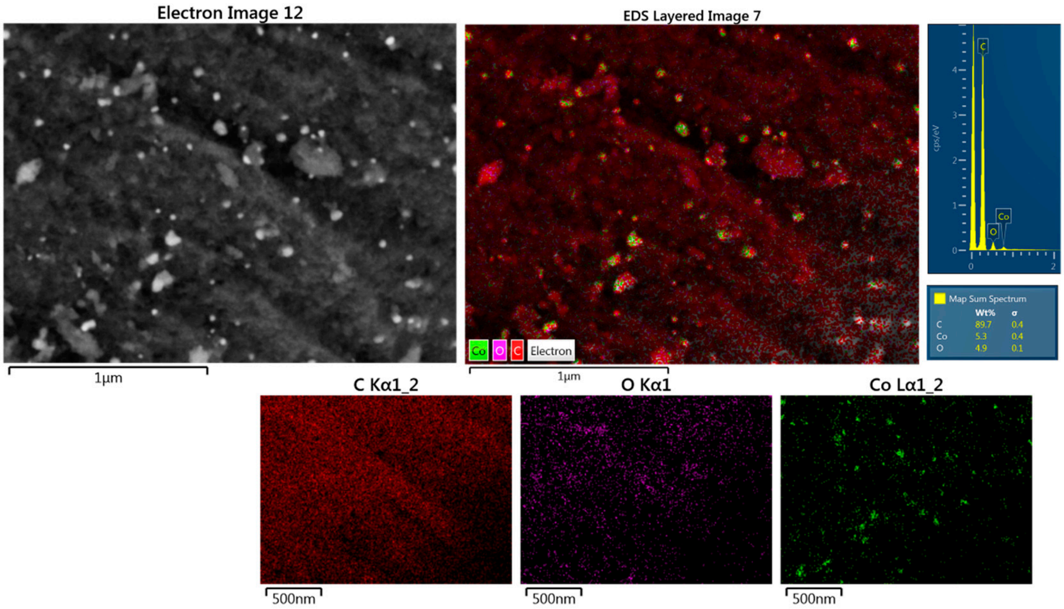

Figure SI Co 5. Elemental mapping of G2N50 Co. With respect to GN50 Co, bigger nanoparticles can be observed, with a broader size distribution. The elemental distribution is very close to 5 wt. %.

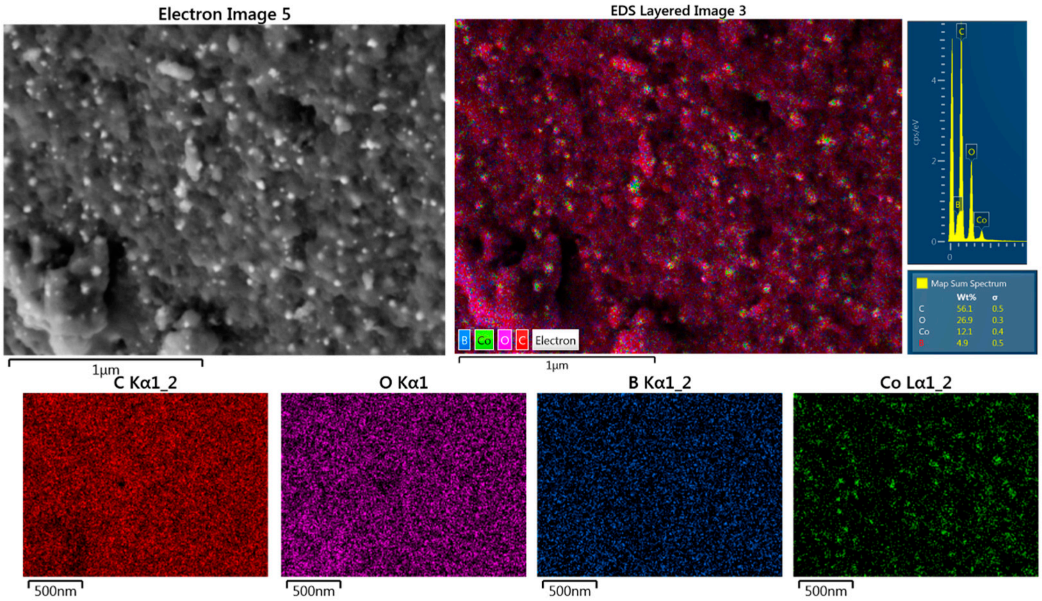

Figure SI Co 6. Elemental mapping of GN50 Co with excess LiBH4. The matrix is wet with LiBH4 derivative, but a molten texture can still be observed, with relatively big nanoparticles of Co visible by transparency. The observed proportion of elemental Co (12.1 wt. %) is higher than expected.
