# Supplementary material for: From Iron to Copper: The Effect of Transition Metal Catalysts on the Hydrogen Storage Properties of Nanoconfined LiBH4 in a Graphene-Rich N-Doped Matrix
Source: Molecules. 2022 May 3;27(9):2921. doi: 10.3390/molecules27092921 (PMC9103407; doi:10.3390/molecules27092921)
Supplement: Supplementary file 1 [file molecules-27-02921-s001.zip › Molecule 2022 SI Cu Map.pdf]

From iron to copper: the effect of transition metal catalysts on the hydrogen storage properties of nanoconfined $LiBH_4$ in a graphene-rich N-doped matrix.

Alejandra A. Martínez [1,2], Aurelien Gasnier [1,2,*] and Fabiana C. Gennari [1,3]

1 Consejo Nacional de Investigaciones Científicas y Técnicas (CONICET) and Centro Atómico Bariloche (CNEA), Av. Bustillo 9500, R8402AGP, S. C. de Bariloche, Río Negro, Argentina; andreaalejandra.m5@gmail.com (A.M.); gennari@cab.cnea.gov.ar (F.G.)
2 Instituto de Nanociencia y Nanotecnología, S. C. de Bariloche, Río Negro, Argentina
3 Instituto Balseiro, Universidad Nacional de Cuyo, Argentina
* Correspondence: aurelien.gasnier@cab.cnea.gov.ar; Tel.: +54-294-444-5556

elemental mapping of Cu-decorated matrixes (SI Cu Map)

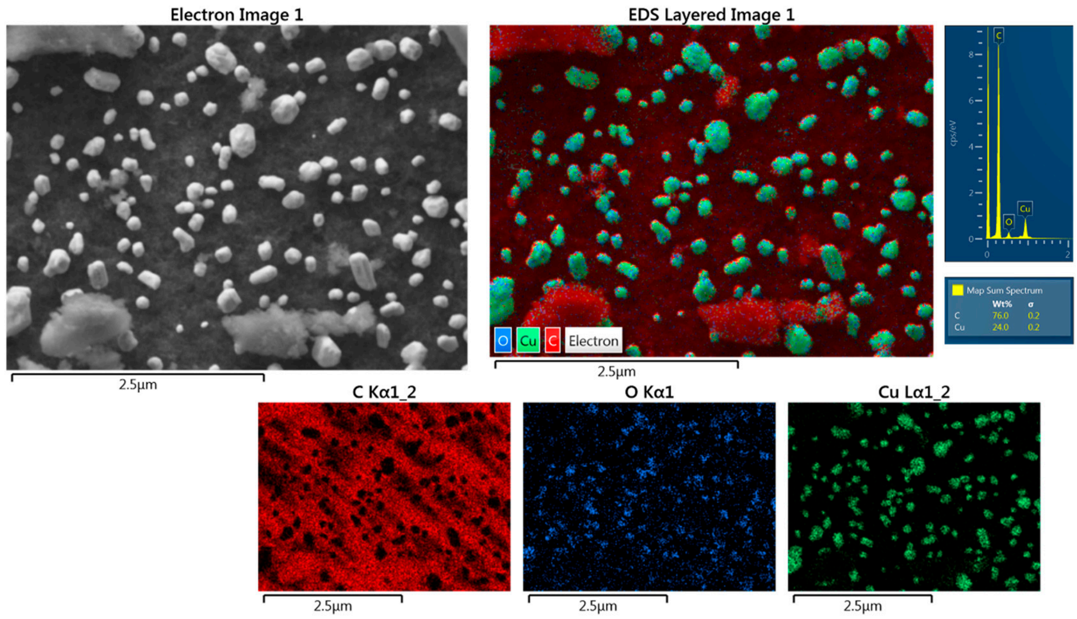

Figure SI Cu 1. Elemental mapping of GN Cu. Big Cu nanoparticles of geometrical shapes are observed, and the elemental distribution of Cu (24.0 wt. %) is higher than expected, suggesting Cu is concentrated at the surface of the matrix.

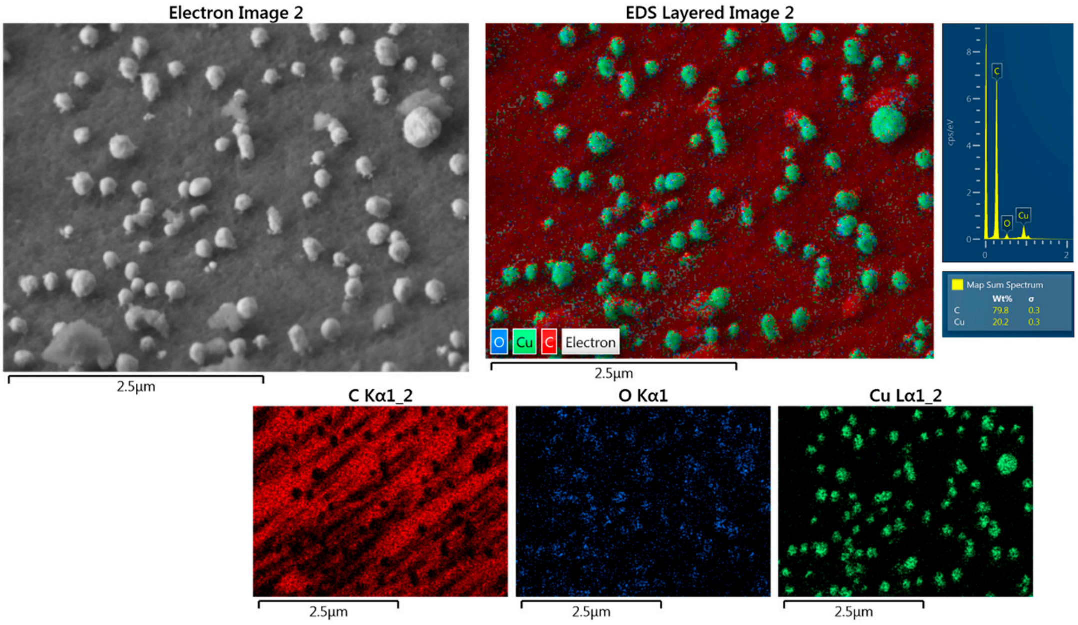

Figure SI Cu 2. Elemental mapping of G2N Cu. Big Cu nanoparticles of less defined shaped are observed, with some rugosity. The high Cu elemental distribution (20.2 wt. %) suggests Cu is concentrated at the surface of the matrix.

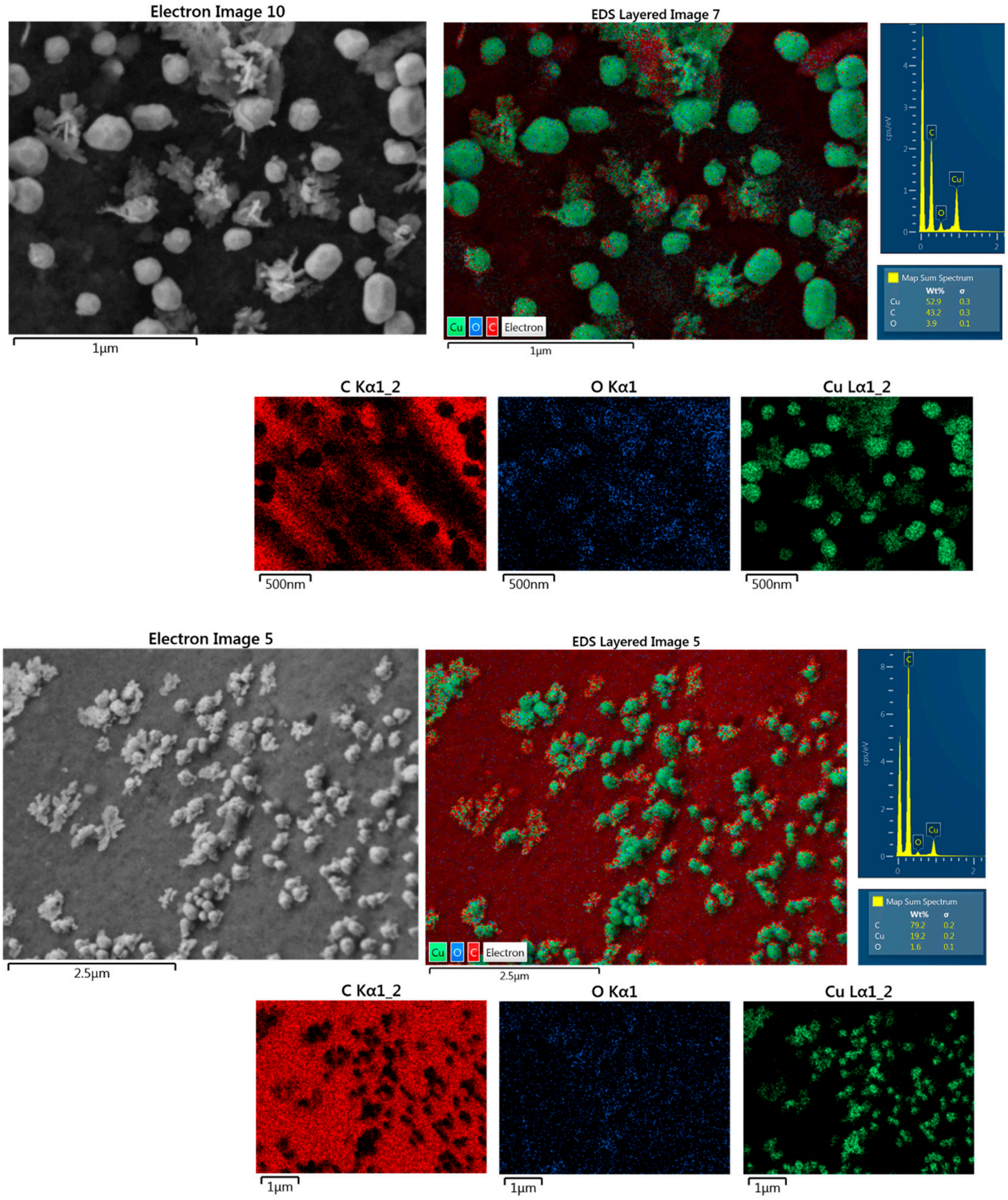

Figure SI Cu 3. Elemental mapping of G2N50 Cu. Big geometric nanoparticles is observed next to more spilled morphologies; both can be attributed to Cu. The proportion of Cu is very important (from 19.2 to 52.9 wt. %), suggesting that Cu is particularly concentrated at the surface of the matrix.

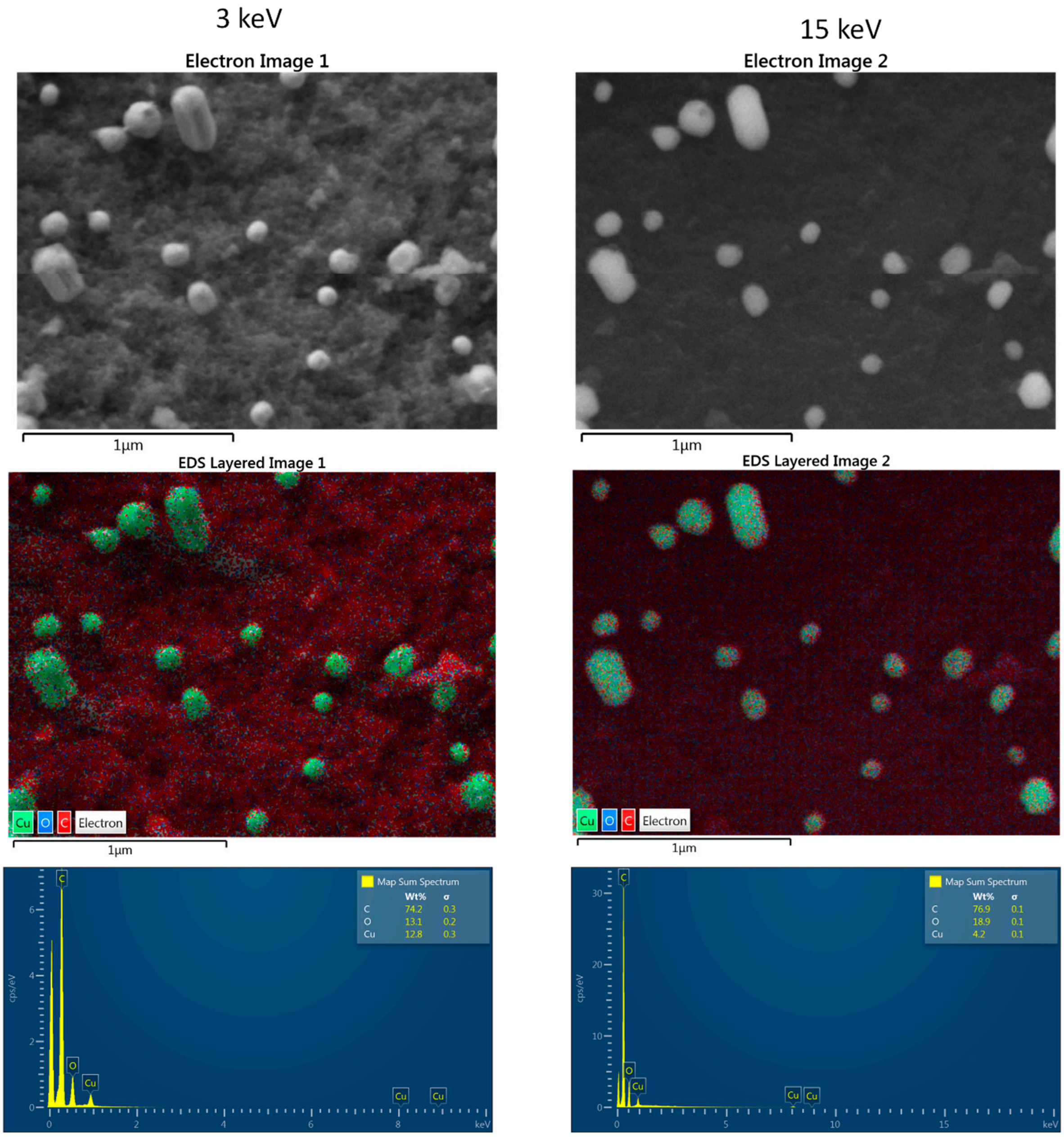

Figure SI Cu 4. Elemental mapping of GN50 Cu at 3 keV (left) and 15 keV (right). Geometric nanoparticles of big size can be observed. The elemental distribution of Cu at 3 keV (12.8 wt. %) is higher than expected but lowers to 4.2 wt. % at 15 keV, a value closer to the expected one (5 wt. %), a sign that copper might be concentrated at the surface of the matrix.

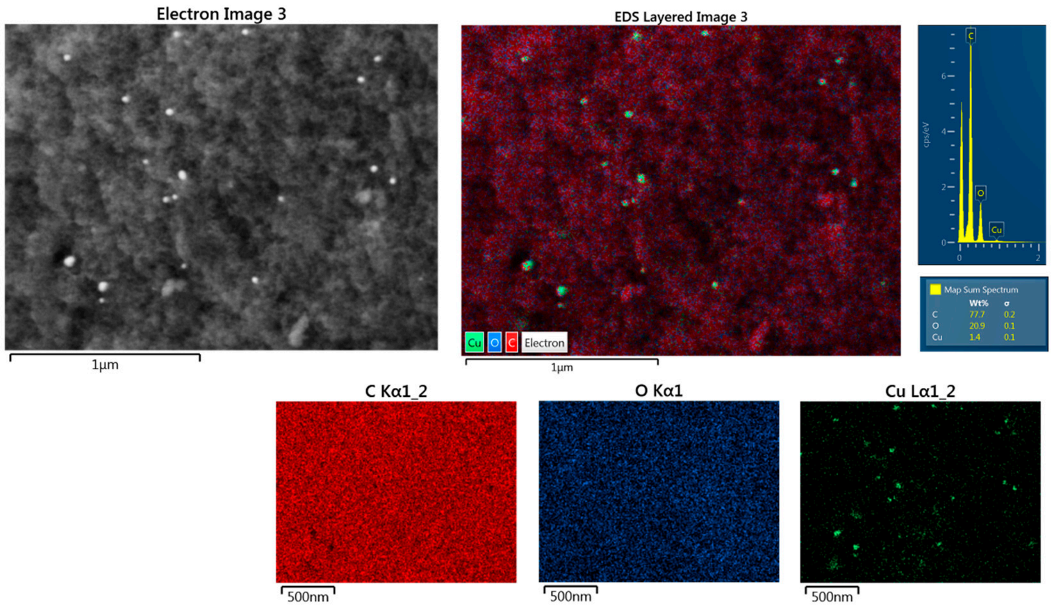

Figure SI Cu 5. Elemental mapping of GN50 Cu. Particles of much smaller size appear in LiBH4 impregnated GN Cu, that can be attributed to elemental Cu. The elemental composition presents low proportion of Cu (1.4 wt. %), suggesting an inhomogeneous repartition of Cu in the sample and a low threshold for Cu particles size growth.

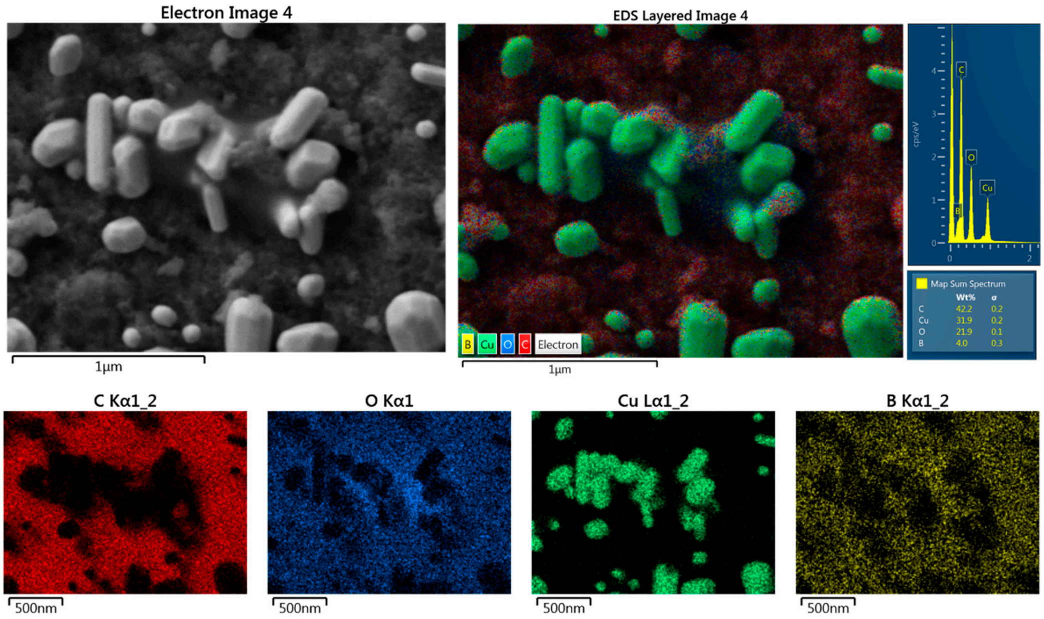

Figure SI Cu 6. Elemental mapping of GN50 Cu with excess LiBH4. Geometrical Cu nanoparticles of big size are concentrated within a spilled degradation product of LiBH4. The Cu elemental proportion (31.9 wt. %) is higher than expected, and B appears to be more concentrated around the Cu aggregate.

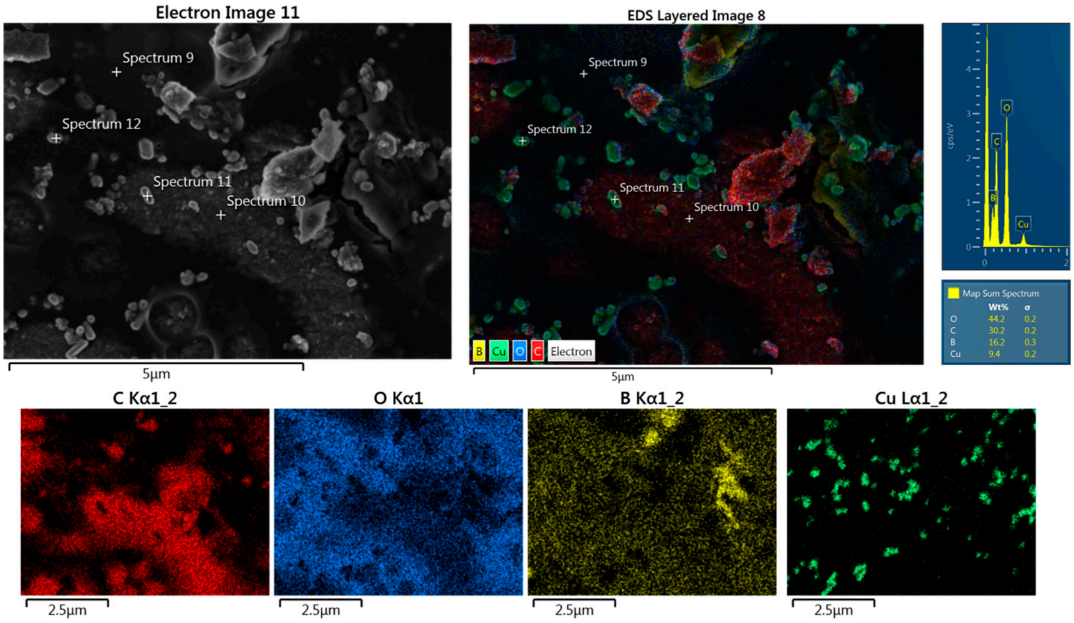

Figure SI Cu 7. Elemental mapping of GN50 Cu with large excess of $LiBH_4$. Cu aggregates seem to be concentrated at the junction between the matrix and the spilled $LiBH_4$ degradation product. B appears to be more concentrated near cracks. The elemental concentration of Cu (9.4 wt. %) is higher than expected (5 wt. %) on this large area, but lower than usually observed for Cu samples (~20 wt. %). The observed proportion of elemental B is much higher (16.2 wt. %) than usual for this kind of samples.
