# Supplementary material for: From Iron to Copper: The Effect of Transition Metal Catalysts on the Hydrogen Storage Properties of Nanoconfined LiBH4 in a Graphene-Rich N-Doped Matrix
Source: Molecules. 2022 May 3;27(9):2921. doi: 10.3390/molecules27092921 (PMC9103407; doi:10.3390/molecules27092921)
Supplement: Supplementary file 1 [file molecules-27-02921-s001.zip › Molecule 2022 SI Fe Map.pdf]

From iron to copper: the effect of transition metal catalysts on the hydrogen storage properties of nanoconfined $LiBH_4$ in a graphene-rich N-doped matrix.

Alejandra A. Martínez [1,2], Aurelien Gasnier [1,2,*] and Fabiana C. Gennari [1,3]

1 Consejo Nacional de Investigaciones Científicas y Técnicas (CONICET) and Centro Atómico Bariloche (CNEA), Av. Bustillo 9500, R8402AGP, S. C. de Bariloche, Río Negro, Argentina; andreaalejandra.m5@gmail.com (A.M.); gennari@cab.cnea.gov.ar (F.G.)
2 Instituto de Nanociencia y Nanotecnología, S. C. de Bariloche, Río Negro, Argentina
3 Instituto Balseiro, Universidad Nacional de Cuyo, Argentina
* Correspondence: aurelien.gasnier@cab.cnea.gov.ar; Tel.: +54-294-444-5556

elemental mapping of Fe-decorated matrixes (SI Fe Map)

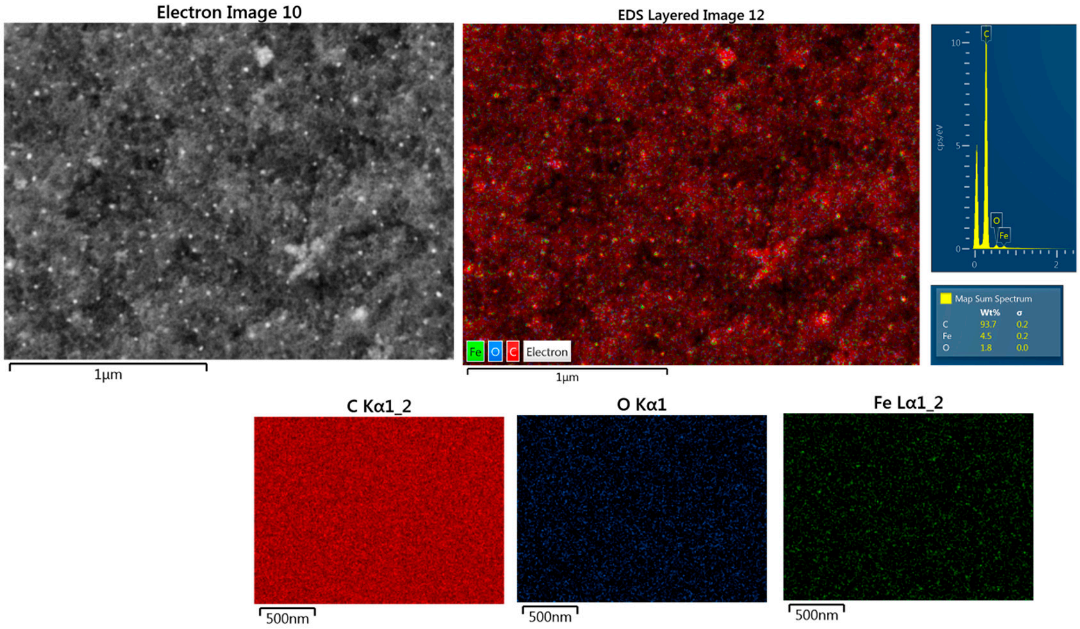

Figure SI Fe 1. GN Fe Map composition. Homogenous distribution of small Fe nanoparticles can be observed, with an elemental contribution of almost 5 wt. %

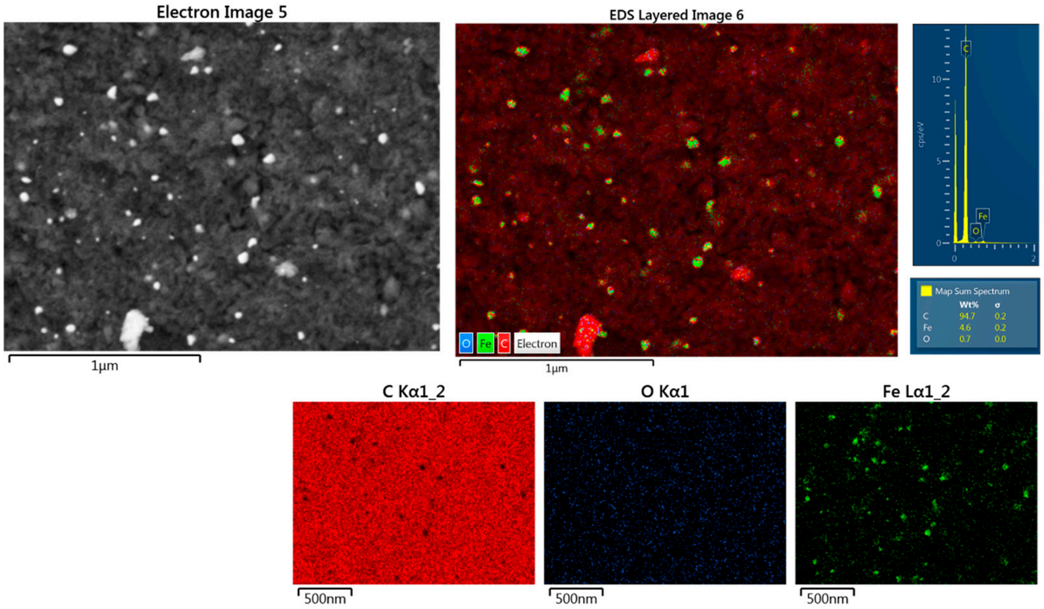

Figure SI Fe 2. G2N Fe Map composition. The distribution of Fe nanoparticles is a bit less homogeneous, in particular their size is a bit bigger with a broader dispersion; elemental contribution of Fe is almost 5 wt. %

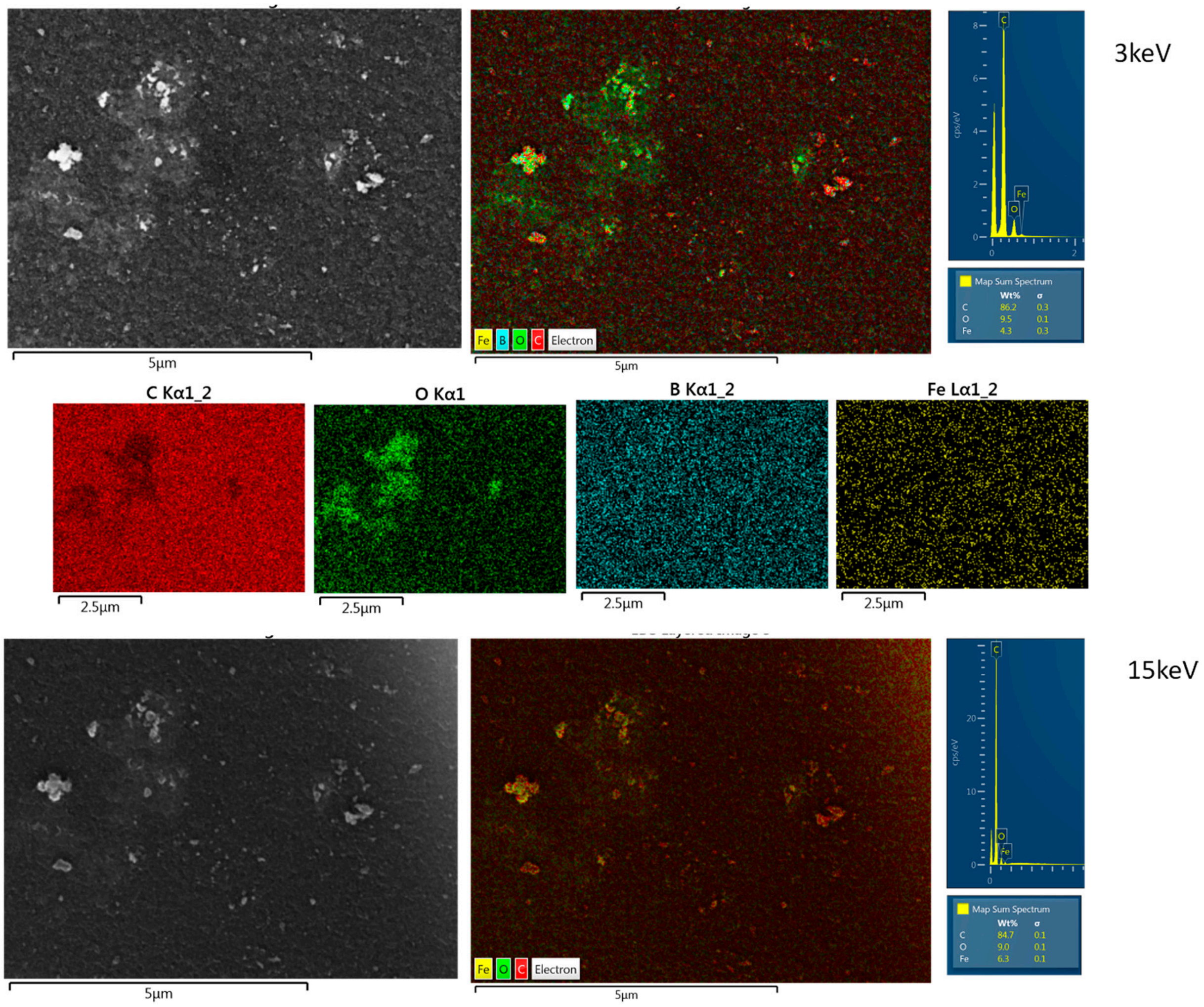

Figure SI Fe 3. Map composition of GN50 Fe at 3 keV (up) versus 15 keV (down). In each case the proportion of elemental iron is similar and close to the expected value (5 wt. %), indicating that the metal nanoparticles are not only located at the surface of the matrix. If Fe was located mostly on the surface of the matrix, its proportion at low energy (3 keV) should be higher as the penetration is shorter (˜50 nm) than for higher energy (1 um at 15 keV).

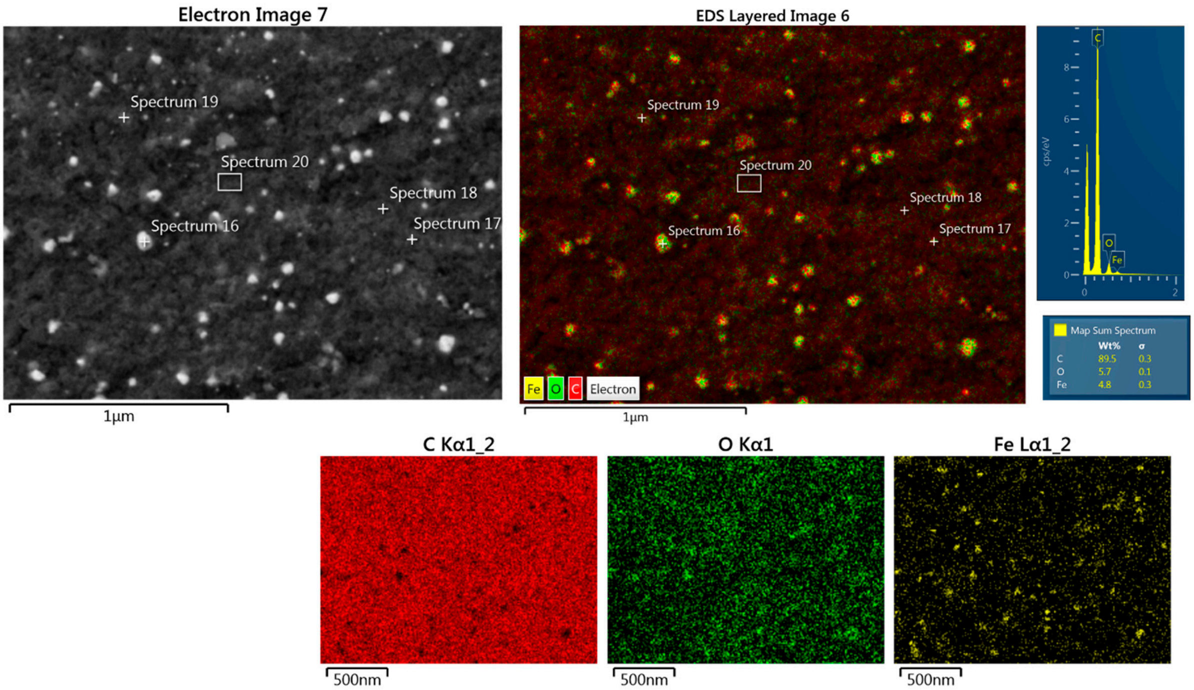

Figure SI Fe 4. Map composition of G2N50 Fe. The proportion of Fe is similar to the expected value and the nanoparticles are bigger than for GN50 Fe.

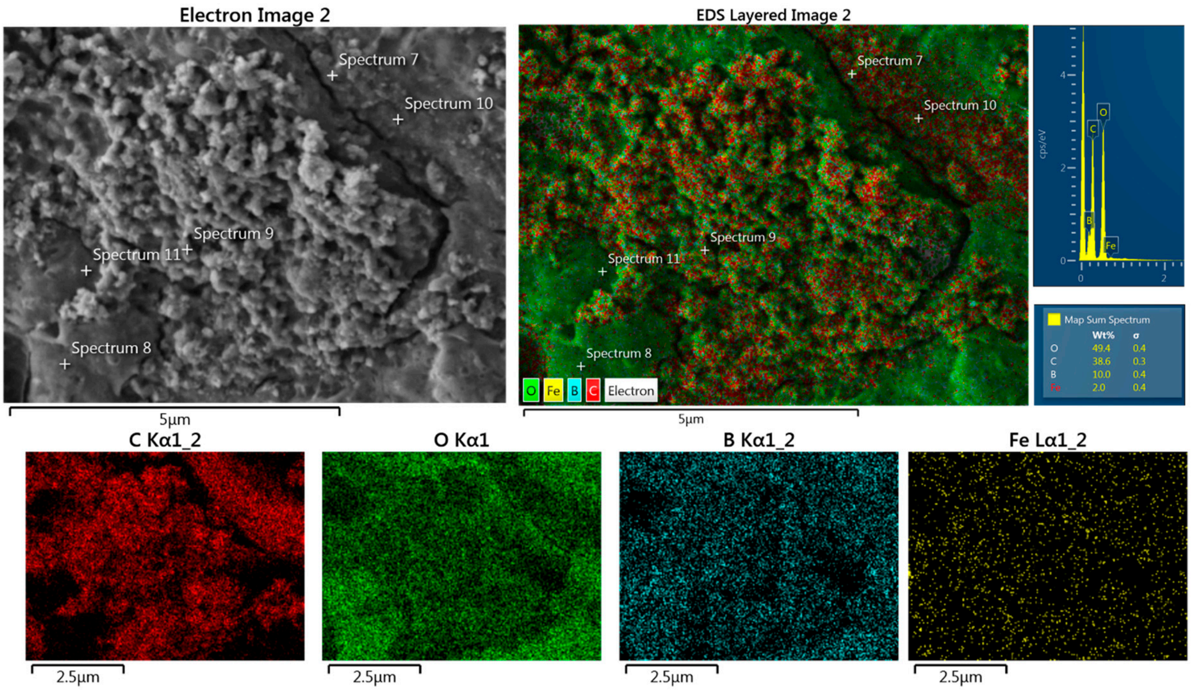

Figure SI Fe 5. Map-composition of GN50 Fe in a zone of excess $LiBH_4$. It indicates excessive proportions of boron and oxygen, and low relative values of iron and carbon (while their respective proportions are in accordance with 5 wt% Fe per mass of C). The oxygen is more present on the corner of the image, where a flat platform with cracks is observed, while more carbon can be observed in the centre of the image, where a more textured material is observed. On the centre the boron derivative is wetting the structure of the carbon matrix while on the edges it is spilling over it.

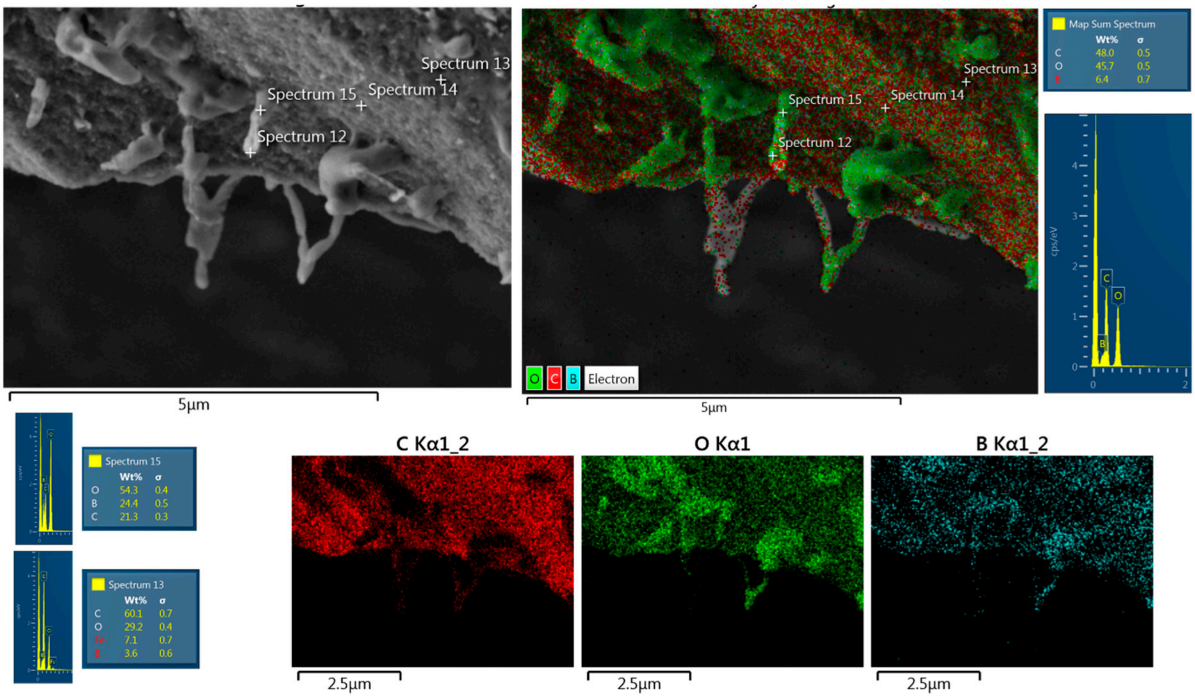

Figure SI Fe 6. Map-composition of GN50 Fe with excess LiBH4. It indicates the strand are composed mostly of boron and oxygen.

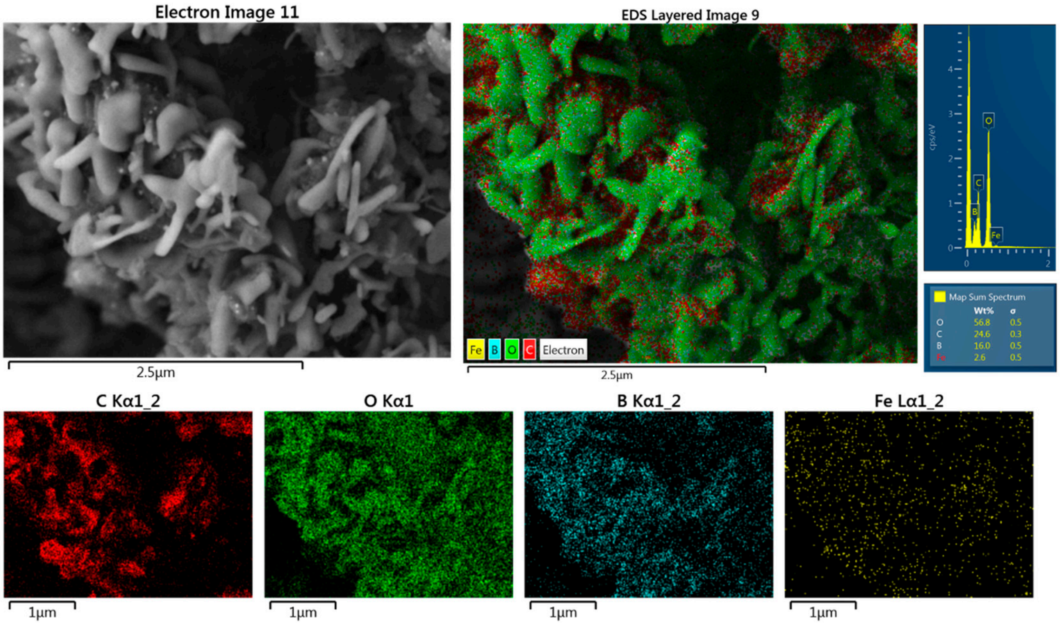

Figure SI Fe 7. Map composition of G2N50 Fe on a zone with excess LiBH4. The flake-like structures bursting out of the matrix are mostly composed of boron and oxygen. Most of these melted upon scanning the zone but some retained their flat, sharp-edged, structure
