# Supplementary material for: From Iron to Copper: The Effect of Transition Metal Catalysts on the Hydrogen Storage Properties of Nanoconfined LiBH4 in a Graphene-Rich N-Doped Matrix
Source: Molecules. 2022 May 3;27(9):2921. doi: 10.3390/molecules27092921 (PMC9103407; doi:10.3390/molecules27092921)
Supplement: Supplementary file 1 [file molecules-27-02921-s001.zip › Molecule 2022 SI Ni Map.pdf]

From iron to copper: the effect of transition metal catalysts on the hydrogen storage properties of nanoconfined $LiBH_4$ in a graphene-rich N-doped matrix.

Alejandra A. Martínez [1,2], Aurelien Gasnier [1,2,*] and Fabiana C. Gennari [1,3]

1 Consejo Nacional de Investigaciones Científicas y Técnicas (CONICET) and Centro Atómico Bariloche (CNEA), Av. Bustillo 9500, R8402AGP, S. C. de Bariloche, Río Negro, Argentina; andreaalejandra.m5@gmail.com (A.M.); gennari@cab.cnea.gov.ar (F.G.)
2 Instituto de Nanociencia y Nanotecnología, S. C. de Bariloche, Río Negro, Argentina
3 Instituto Balseiro, Universidad Nacional de Cuyo, Argentina
* Correspondence: aurelien.gasnier@cab.cnea.gov.ar; Tel.: +54-294-444-5556

elemental mapping of Ni-decorated matrixes (SI Ni Map)

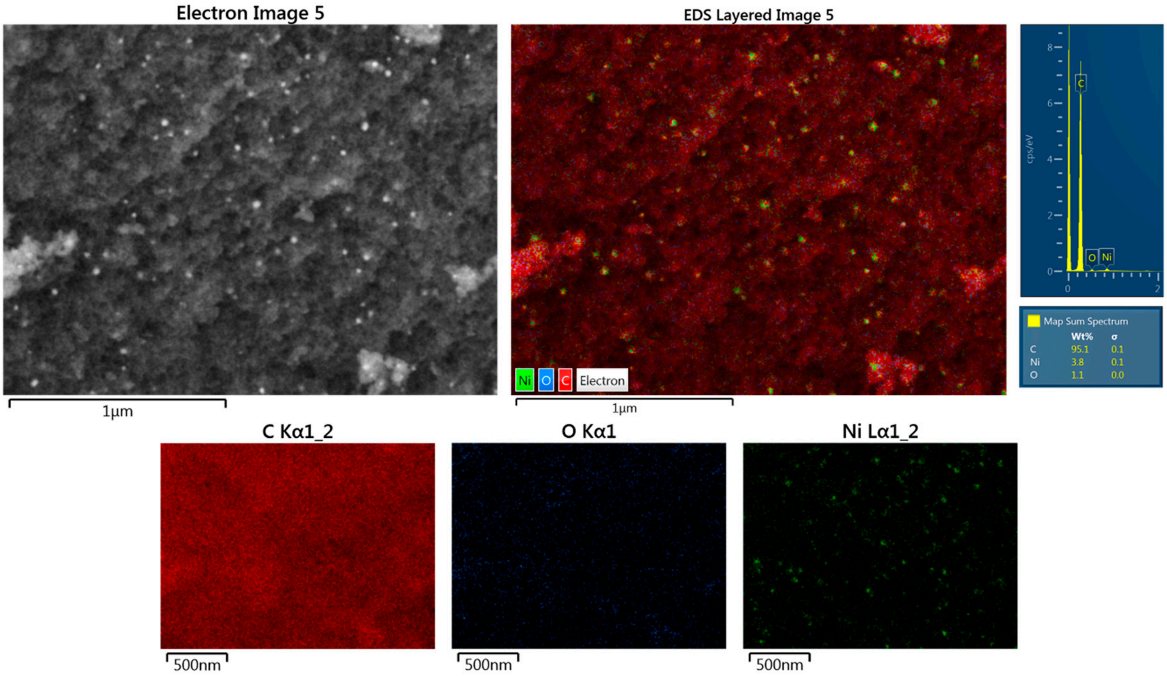

Figure SI Ni 1. Elemental mapping of GN Ni. Small to medium nanoparticles of Ni are observed. Elemental repartition of Ni is a bit low (3.8 wt. %).

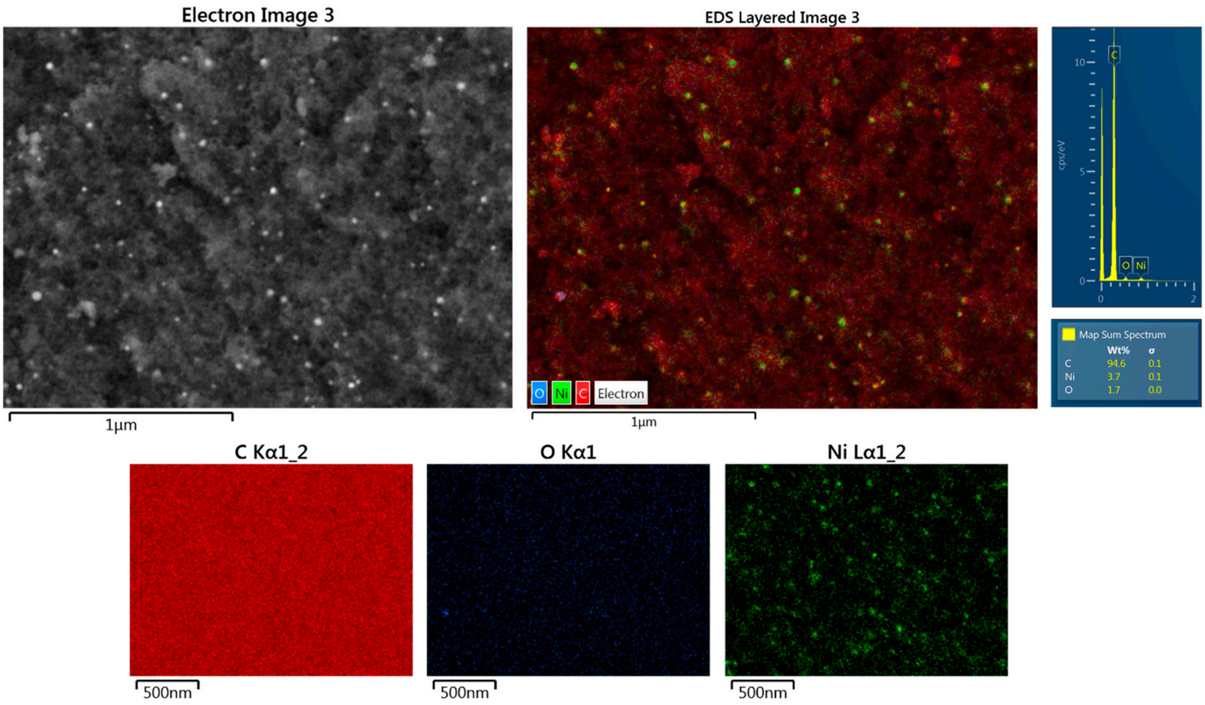

Figure SI Ni 2. Elemental mapping of G2N Ni. Small to medium nanoparticles of Ni are observed. Elemental repartition of Ni is a bit low (3.8 wt. %).

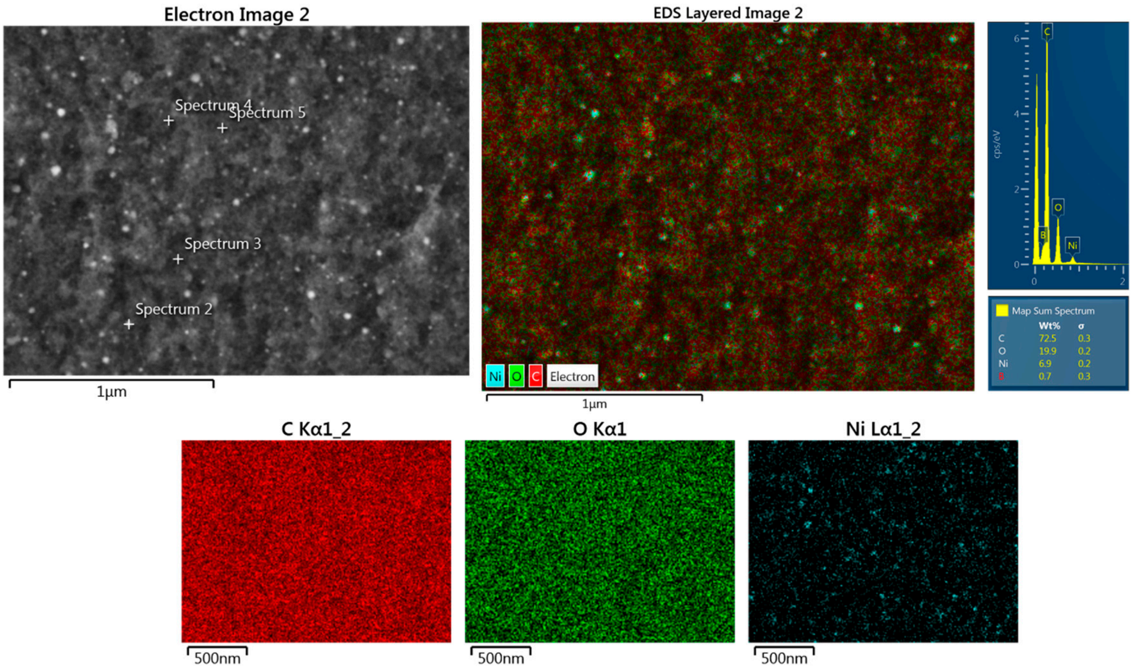

Figure SI Ni 3. Elemental mapping of GN50 Ni. Small to medium nanoparticles of Ni are observed, with elemental repartition (6.9 wt. %) a bit higher than expected.

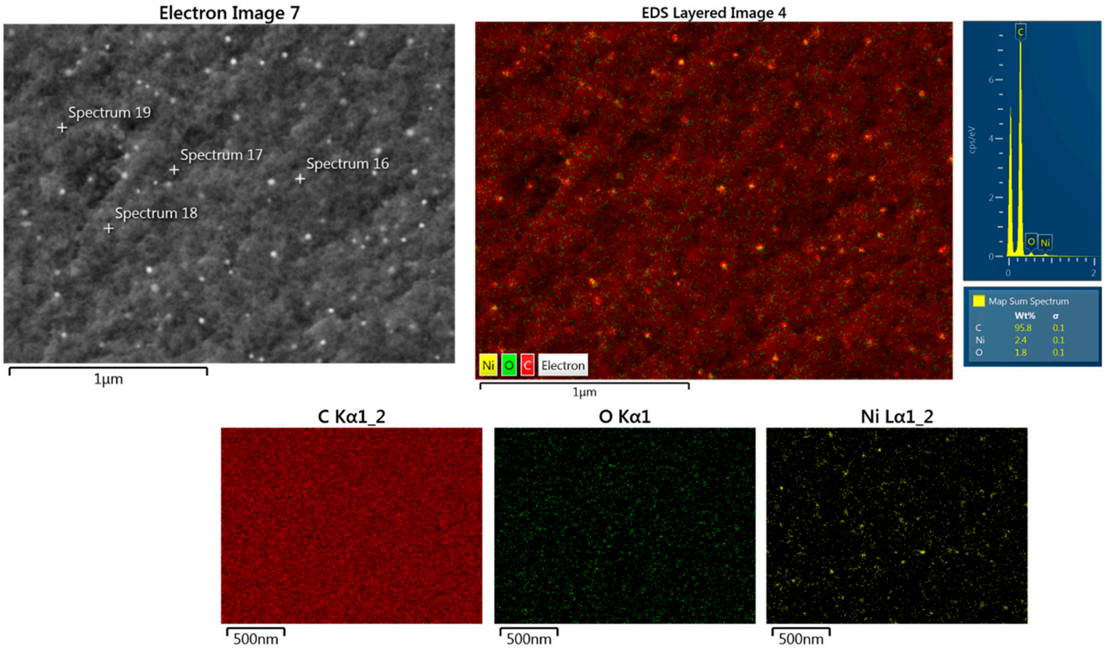

Figure SI Ni 4. Elemental mapping of GN50 Ni. Medium nanoparticles of Ni are observed, with elemental repartition (2.4 wt. %) quite lower than expected.
